# Gender-specific association of *STON2* rs2371597 polymorphism in keratoconus patients of Saudi origin

**DOI:** 10.3389/fgene.2024.1505629

**Published:** 2024-12-09

**Authors:** Altaf A. Kondkar, Tahira Sultan, Taif A. Azad, Tanvir Khatlani, Glenn P. Lobo, Hatem Kalantan, Saleh A. Al-Obeidan, Abdulrahman M. Al-Muammar

**Affiliations:** ^1^ Department of Ophthalmology, College of Medicine, King Saud University, Riyadh, Saudi Arabia; ^2^ Glaucoma Research Chair in Ophthalmology, College of Medicine, King Saud University, Riyadh, Saudi Arabia; ^3^ King Saud University Medical City, King Saud University, Riyadh, Saudi Arabia; ^4^ Department of Blood and Cancer Research, King Abdullah International Medical Research Center, King Saud Bin Abdulaziz University of Health Sciences, Ministry of National Guard Health Affairs, Riyadh, Saudi Arabia; ^5^ Department of Ophthalmology and Visual Neurosciences, University of Minnesota, Minneapolis, MN, United States

**Keywords:** case-control study, gender differences, genetic polymorphisms, keratoconus, STON2, PDIA5, WNT1, ABCA6

## Abstract

**Objective:**

To investigate the association of specific genetic polymorphisms (rs2371597 in *STON2*, rs11720822 in *PDIA5*, rs387907358 in *WNT1*, and rs77542162 in *ABCA6*) in a Saudi cohort of keratoconus (KC) patients compared to controls.

**Methods:**

A retrospective case-control genetic association study was conducted. The study included 99 KC patients and 193 healthy controls. Genotyping was performed using real-time PCR with TaqMan assays. Associations between genetic polymorphisms and KC were assessed using various genetic models and binary logistic regression analysis.

**Results:**

None of the tested polymorphisms showed an overall association with KC risk. Specifically, the rs2371597 polymorphism in *STON2* did not demonstrate a significant association with KC risk across different genetic models. However, a gender-specific effect of rs2371597 was noted: in men, the C/G genotype was associated with a higher risk of KC, particularly in the dominant model, while no significant association was observed in women. Age and sex were identified as significant predictors of KC risk, but rs2371597 did not significantly affect KC risk in regression analysis.

**Conclusion:**

Preliminary evidence suggests a gender-specific effect of the rs2371597 polymorphism in *STON2*, with an increased KC risk associated with C/G-C/C genotypes in men which was age-dependent. This result highlights the importance of considering population-specific genetic factors and the potential gender-specific effects on KC susceptibility. However, these findings need further validation with larger age- and sex-matched samples of diverse populations.

## Introduction

Keratoconus (KC) is a progressive eye condition characterized by the gradual thinning and protrusion of the cornea, which distorts its normally round shape into a cone-like structure. This deformation leads to significant visual disturbances, including blurred vision, increased sensitivity to glare, and irregular astigmatism (Rabinowitz, 1998). Globally, KC affects approximately 1 in 2,000 individuals. However, prevalence rates can vary based on geographic and demographic factors. In Saudi Arabia, KC is relatively common, with estimates suggesting that it affects between 1 in 375 and 1 in 1,000 people manifesting between the early teenage years and young adulthood in most studies (Torres Netto et al., 2018; Alzahrani et al., 2021; Gordon-Shaag et al., 2015; Godefrooij et al., 2017). This higher prevalence might be attributed to specific genetic and environmental influences in the region.

The exact etiology of KC is not fully understood, but it is believed to involve a combination of environmental, biochemical and genetic factors highlighting the complex multifactorial nature of KC (Ferrari and Rama, 2020). Environmental influences such as sun exposure and mechanical stress (e.g., eye rubbing) are also associated with KC (Gordon-Shaag et al., 2015). Sex hormones have been implicated in KC and it has been noted that KC develops earlier and progresses more rapidly in men than women (Fink et al., 2005), potentially interacting with genetic predispositions to influence disease risk (Meng and Ren, 2024). There is significant interest in the genetic basis of KC because it is innate in families, indicating a hereditary component. Several genomic studies have highlighted the potential role of genetic mutations and polymorphisms in contributing to disease susceptibility and the pathogenesis of KC (Hao et al., 2021; Bykhovskaya and Rabinowitz, 2021; Song et al., 2024; Wonneberger et al., 2024; Khashim Alswailmi et al., 2023). Despite extensive research, the precise genetic determinants of KC remain elusive, making it a subject of considerable scientific interest. Investigating the genetic variants in KC is crucial to enhance our understanding of the molecular mechanisms underlying the disease and for developing potential genetic markers for early diagnosis and targeted therapeutic strategies.

Stonin 2 (*STON2*) gene encodes a protein crucial for intracellular transport processes, including clathrin-mediated endocytosis and vesicular trafficking. Though mainly recognized for its role in cellular transport, alterations in STON2 function can potentially contribute to neurodegenerative disorders (Luan et al., 2011; Ma et al., 2024; Mahapatra et al., 2023; Xu et al., 2018) and cancer (Mahapatra et al., 2023; Xu et al., 2018). Studies have found that the SNP rs2371597 in the *STON2* gene is associated with an increased risk of developing KC and might influence cellular functions relevant to corneal structure and integrity. The polymorphism rs2371597 has been strongly associated with CCT and KC development in the Japanese and Han Chinese population (Hosoda et al., 2020; Zhang et al., 2021). Still, it has not been investigated in other ethnicities, including the Arabs of Saudi origin.

The rs11720822 polymorphism in the *PDIA5* gene is associated with glaucoma due to its role in the unfolded protein response (UPR) and endoplasmic reticulum (ER) stress (Carbone et al., 2011; Ayub et al., 2014). The *PDIA5* gene, encoding protein disulfide isomerase family A, member 5, is involved in protein folding and maintenance of cellular homeostasis (Villani et al., 2012). Given the overlap in pathophysiological mechanisms between glaucoma and keratoconus, including cellular stress and matrix integrity, this genetic variant might impact KC by affecting similar underlying processes. There are no studies directly linking the rs11720822 *PDIA5* polymorphism to KC.

The *WNT1* gene is a critical component of the Wnt signaling pathway, which regulates cell proliferation, differentiation, and migration (Yu et al., 2024). Disruptions in the Wnt pathway have been associated with various ocular diseases (Nguyen et al., 2022; Karolak et al., 2020a; Wang et al., 2024). Karolack et al. reported an accumulation of sequence variants in the Wnt signaling pathway and identified a missense variant rs387907358 (c.1063G > T, p.(V355F)) in *the WNT1* gene in corneal tissue of Polish KC patients suggesting a potential role in KC (Karolak et al., 2020b). In addition, the *ABCA6* gene, involved in lipid transport and metabolism, has been associated with various metabolic disorders (Kaminski et al., 2001; van Leeuwen et al., 2015). The rs77542162 variant in *ABCA6* was reported to be associated with corneal biochemical properties and KC in the Europeans, suggesting its potential role in altering corneal biomechanics and structure and, thereby, in KC development (Khawaja et al., 2019).

Since the genetic etiology of KC is heterogeneous with multiple genes underlying its pathogenesis, the study aimed to investigate the association of these specific genetic polymorphisms (rs2371597 in *STON2*, rs11720822 in *PDIA5*, rs387907358 in *WNT1*, and rs77542162 in *ABCA6*) with KC in a cohort of Saudi patients. By focusing on Middle Eastern Arabs of Saudi origin, this study seeks to provide insights into the genetic basis of KC in a demographic with a unique genetic background, potentially revealing genetic risk factors and contributing to understanding this complex ocular disease in this ethnicity.

## Materials and methods

### Study population and design

Our retrospective case-control genetic association study adhered to the Declaration of Helsinki guidelines. We obtained written informed consent from all participants, and ethical approval from the College of Medicine Institutional Review Board (proposal number #09–659) at King Saud University in Riyadh, Saudi Arabia.

Participants included (n = 99) patients diagnosed with Keratoconus (KC) at the anterior segment clinic of King Abdulaziz University Hospital, Riyadh (Abu-Amero et al., 2014). The diagnosis was confirmed based on established clinical criteria: a Schimpff-flow-based elevation map demonstrating posterior corneal elevation within the central 5 mm of ≥ +20 μm, an inferior-to-superior (I-S) dioptric asymmetry value exceeding 1.2 diopters (D), and a steepest keratometry measurement more significant than 47 D. All participants were unrelated. Individuals were excluded if they had secondary KC resulting from trauma, surgery, Ehlers-Danlos syndrome, osteogenesis imperfecta, pellucid marginal degeneration, or if they had undergone laser-assisted *in situ* keratomileusis (LASIK) resulting in ectasia. Healthy control subjects (n = 193) were selected from the general ophthalmology clinic, all of the Saudi nationality, and bore no ocular diseases or history of ophthalmic surgeries. Each control participant’s corneas were clear upon examination, and their Schimpff-flow-based elevation maps displayed normal findings. Control subjects who opted not to participate were also excluded from the study (Abu-Amero et al., 2014).

### DNA preparation and genotyping

Peripheral EDTA blood was used for DNA extraction using the QIAamp DNA Mini Kit as per the manufacturer’s instructions (Cat. No. 51306, Qiagen, Hilden, Germany). For genotyping, commercially available TaqMan^®^ genotyping assay mix (Cat. No.: 4331349; Applied Biosystems Inc., Foster City, CA, USA) was used–rs2371597 in *STON2* (Assay ID: C___2791741_10), rs11720822 in *PDIA5* (C__11238237_10), rs387907358 in *WNT1* (C_322377343_10), and rs77542162 in *ABCA6* (C_102267712_10) according to the manufacturer’s protocol for real-time PCR (ABI-7500, Applied Biosystems) as described previously (Kondkar et al., 2022). Genotype calling was performed using the allele discrimination software version 2.0.5 in ABI-7500. Polymorphism details are shown in Table 1.

**TABLE 1:** Details of the polymorphisms investigated in this study.

SNP ID	Gene	Location on Build GRCh38	SNP context sequence [VIC/FAM]
rs2371597	*STON2*	Chr.14:81407033	ACT​CCA​GCT​GAC​CAC​ACT​GCA​GTC​A[C/G]GAA​ACT​CTT​CCC​TGG​AAA​AGA​GCC​A
rs11720822	*PDIA5*	Chr.3:123150194	GAT​TTC​CTT​GCC​CAT​CTA​AAA​ATC​T[C/T]TCC​TTA​TGG​CTG​CCT​C
rs387907358	*WNT1*	Chr.12:48981590	CTG​CAC​CTT​CCA​CTG​GTG​CTG​CCA​C[G/T]TCA​GCT​GCC​GCA​ACT​GCA​CGC​ACA​C
rs77542162	*ABCA6*	Chr.17:69085137	GGC​CAC​AGC​ACG​TTC​TCT​TGA​GGG​C[A/G]GTA​CCC​CAG​GTG​GCC​CAA​AAC​TGA​A

### Statistical analysis

For the current study, continuous variables were analyzed using the Mann–Whitney U-test following normality assessments by the Kolmogorov–Smirnov test. The Hardy–Weinberg equilibrium (HWE) deviation and genetic associations with KC was tested using Chi-square and Fisher’s exact tests as appropriate. The influence of risk factors—including age, sex, and genotype—on KC was assessed through binary logistic regression analysis. All statistical analyses were conducted using SPSS software, version 25 (IBM Inc., Chicago, IL, USA) and SNPStats online version 1.0, accessible at SNPStats (https://www.snpstats.net/start.htm accessed on 14 August 2024). Power calculation was performed using the stand-alone PS program version 3.1.6. A significant threshold p-value was set at less than 0.05. Risk estimates were reported as odds ratios (OR) with 95% confidence intervals (CI).

## Results

The demographic analysis revealed a notable age difference between the control and KC patient groups, with controls being significantly older, ranging from 35 to 75 years (average age 59.0 years) compared to younger KC patients ranging from 12 to 45 years (average age 25.9 years), as indicated by a p-value of <0.001 (Table 2). In contrast, the gender distribution between the two study groups was similar and showed no significant difference (*p* = 0.3142) (Table 2).

**TABLE 2:** Demographic characteristics and distribution of minor allele frequency of investigated polymorphisms in Keratoconus patient and control participants.

Characteristics	Controls (n = 193)	KC (n = 99)	Odds ratio (95% confidence interval)	p-value	HWE p-value
Age in years (SD)	59.0 (10.3)	25.9 (9.1)	—	<0.001^a^	—
Male/Female, n	119/74	55/44	—	0.314^b^	—
Minor Allele Frequency					
rs2371597 [C]	0.38	0.39	1.05 (0.74–1.50)	0.764^c^	0.270^b^
rs11720822 [T]	0.02	0	—	0.101^c^	1.000^b^
rs387907358 [T]	0	0	—	—	—
rs77542162 [G]	0	0	—	—	—

^a^ Mann-Whitney U-test.

^b^ Chi-square test.

^c^ Fisher Exact Probability Test, HWE, Hardy-Weinberg Equilibrium.

Regarding the minor allele frequencies (MAFs) and their association with KC, none of the tested polymorphisms showed an allelic connection to the disease (Table 2). The allele frequency for rs2371597 [C] in *STON2* was similar between controls (0.38) and KC patients (0.39), and the odds ratio (1.05) with a p-value of 0.7643 suggests no significant association with KC. The rs11720822 [T] variant in *PDIA5* showed a low frequency in controls (0.02) and was absent in KC patients with a p-value of 0.1013, indicating no potential association with KC. Also, rs387907358 [T] and rs77542162 [G] variants in *WNT1* and *ABCA6* were absent in either group. Hence, further statistical analysis was performed only for rs2371597 *STON2* polymorphism. Overall, these results imply that the allele frequency of the studied polymorphisms is not associated with KC.

The overall genotype analysis of the rs2371597 polymorphism in the *STON2* gene showed that this genetic variant is not significantly associated with KC risk across different genetic models (Table 3). In the codominant model, the odds ratios for genotypes G/G, C/G, and C/C were close to 1, indicating no notable difference in KC risk compared to the wild-type G/G genotype. Similarly, the dominant model (G/G vs C/G-C/C) and recessive model (G/G-C/G vs C/C) also showed no significant associations, with p-values greater than the significant threshold of 0.05.

**TABLE 3:** Genotype association analysis of polymorphisms rs2371597 in *STON2* with the risk of keratoconus compared to controls under different genetic models and according to gender.

SNP rs2371597	Genetic model	Genotype*	Control n (%)	KC n (%)	Odds ratio (95% confidence interval)	*p*-value^§^	*p*-value^§†^
Overall	Codominant	G/G	78 (40.4)	37 (37.4)	1.00	0.84	0.74
		C/G	83 (43)	46 (46.5)	1.17 (0.69–1.99)		
		C/C	32 (16.6)	16 (16.2)	1.05 (0.51–2.16)		
	Dominant	G/G	78 (40.4)	37 (37.4)	1.00	0.61	0.45
		C/G-C/C	115 (59.6)	62 (62.6)	1.14 (0.69–1.87)		
	Recessive	G/G-C/G	161 (83.4)	83 (83.8)	1.00	0.93	0.88
		C/C	32 (16.6)	16 (16.2)	0.97 (0.50–1.87)		
Men	Codominant	G/G	49 (41.2)	14 (25.4)	1.00	0.093	0.72
		C/G	50 (42)	32 (58.2)	2.24 (1.07–4.70)^‡^		
		C/C	20 (16.8)	9 (16.4)	1.57 (0.59–4.22)		
	Dominant	G/G	49 (41.2)	14 (25.4)	1.00	0.042	0.49
		C/G-C/C	70 (58.8)	41 (74.5)	2.05 (1.01–4.16)		
	Recessive	G/G-C/G	99 (83.2)	46 (83.6)	1.00	0.94	0.87
		C/C	20 (16.8)	9 (16.4)	0.97 (0.41–2.29)		
Women	Codominant	G/G	29 (39.2)	23 (52.3)	1.00	0.33	0.12
		C/G	33 (44.6)	14 (31.8)	0.53 (0.23–1.23)		
		C/C	12 (16.2)	7 (15.9)	0.74 (0.25–2.17)		
	Dominant	G/G	29 (39.2)	23 (52.3)	1.00	0.17	0.06
		C/G-C/C	45 (60.8)	21 (47.7)	0.59 (0.28–1.25)		
	Recessive	G/G-C/G	62 (83.8)	37 (84.1)	1.00	0.96	0.91
		C/C	12 (16.2)	7 (15.9)	0.98 (0.35–2.70)		

* Tested by SNPStat;^‡^G/G vs C/G p-value was significant (Chi-square = 4.64, df = 1, *p* = 0.031); ^§^ Chi-square analysis; ^†^ p-value adjusted for age and sex in overall and for age in men and women groups.

In contrast, gender-specific genotype analyses revealed an interesting finding. In men, the C/G genotype was associated with a significantly increased risk of KC, particularly evident in the dominant model (OR 2.05, *p* = 0.042), suggesting a potential gender-specific effect (Table 3). However, women showed no association between rs2371597 and KC risk across any genetic model. However, there were non-significant trends towards decreased risk with the C/G genotype in the codominant and dominant models. These results imply that while rs2371597 may not broadly impact KC risk, it could have a gender-specific influence in men, warranting further investigation.

The binary logistic regression analysis (Table 4) showed that age was a significant predictor of KC risk. Age was inversely related to KC risk, with each additional year of age associated with a 19% decrease in risk, as indicated by an odds ratio of 0.81 and a highly significant p-value of 0.000. However, it is noteworthy that since KC typically manifests during puberty or early adulthood, the KC participants were significantly younger than the controls. Likewise, sex was also a significant predictor of KC, with females having about 75% lower odds of developing KC compared to males, as reflected in the odds ratio of 0.25 and a p-value of 0.035. In contrast, the rs2371597 polymorphism showed no significant association with KC risk in the codominant or dominant models. The OR for the C/G and C/C genotypes and the combined model of C/G + C/C versus G/G were close to 1 with non-significant p-values of 0.455, 0.678, and 0.451 respectively. This finding suggests that the variant rs2371597 does not significantly influence the risk of KC and the observed gender-effect of the polymorphism in men is likely to be dependent on age. Thus, an age- and sex-matched case-control study in a large cohort would be needed to confirm these findings.

**TABLE 4:** Binary logistic regression analysis to determine the effect of age, sex, and polymorphisms rs2371597 in codominant and dominant models on the risk of keratoconus.

Group variables	B	SE	Wald	Odds ratio (95% confidence interval)	p-value
Age	−0.207	0.027	60.900	0.81 (0.77–0.85)	0.000
Sex	−1.361	0.646	4.438	0.25 (0.07–0.91)	0.035
rs2371597			0.580		0.748
C/G	−0.484	0.647	0.558	0.61 (0.17–0.22)	0.455
C/C	−0.382	0.922	0.172	0.68 (0.11–4.15)	0.678
C/G + C/C	−0.459	0.610	0.568	0.63 (0.19–2.08)	0.451

## Discussion

This study investigated the association of specific genetic polymorphisms in the *STON2*, *PDIA5*, *WNT1*, and *ABCA6* genes with KC in a Saudi cohort. While our findings did not reveal strong overall associations between these polymorphisms and KC risk, we identified a noteworthy gender-specific effect linked to the rs2371597 polymorphism in *STON2*.

KC, characterized by progressive corneal thinning and distortion, poses significant visual impairment challenges. The heterogeneous nature of its etiology suggests that genetic factors may vary widely across populations (Hao et al., 2021; Bykhovskaya and Rabinowitz, 2021). Previous studies, particularly in Japanese and Han Chinese cohorts, reported a strong association between the *STON2* rs2371597 polymorphism and KC (Hosoda et al., 2020; Zhang et al., 2021). Our findings, however, highlight a different dynamic in the Saudi population. The MAF of rs2371597(C) in the KC Saudi cohort (0.39) was slightly higher than the Japanese (0.30) and Han Chinese (0.35). Although we did not observe a general association with KC across the cohort, the gender-specific result in men indicates that this polymorphism may contribute to susceptibility in specific subgroups. Specifically, men with the C/G genotype exhibited a significantly higher risk of developing KC, suggesting potential interactions between genetic and hormonal factors that influence disease manifestation. For example, androgens have been shown to impact corneal healing and epithelial integrity, which may render males more susceptible to developing KC when coupled with genetic predispositions (McKay et al., 2022; Nuzzi and Caselgrandi, 2022). Furthermore, lifestyle factors and environmental exposures may differ by sex, potentially affecting the manifestation of genetic risk factors.

The rs2371597 polymorphism in *STON2* may influence KC through several biological mechanisms. STON2 is involved in intracellular transport processes, including clathrin-mediated endocytosis and vesicular trafficking (Willox and Royle, 2012; Jung et al., 2007). Disruption in these processes could affect cellular homeostasis and corneal cell function. Notably, the involvement of microRNA (miRNA), miRNA-875-3p, in regulating *STON2* expression could also play a crucial role (Wang et al., 2020). MiRNAs can modulate gene expression post-transcriptionally, potentially affecting corneal integrity and susceptibility to KC (Zhang et al., 2022). For instance, the altered expression of STON2, potentially influenced by both genetic factors like the rs2371597 polymorphism and regulatory miRNAs such as miRNA-875-3p, might lead to impaired endocytic processes, exacerbating corneal cell stress and contribute to KC pathogenesis (Hao et al., 2021).

Additionally, STON2 interacts with various signaling pathways, including those related to cellular stress responses (Ma et al., 2024; Mahapatra et al., 2023; Xu et al., 2018). Given the shared pathological features between KC and other ocular conditions, alterations in STON2 could intersect with signaling pathways involved in keratocyte apoptosis and extracellular matrix remodeling, which are pivotal in KC development (Hao et al., 2021). Given the complex etiology of KC, it is possible that different environmental factors might interact with genetic predispositions and increase KC risk (Bykhovskaya and Rabinowitz, 2021). Accordingly, it can be speculated that abnormal collagen synthesis due to hormonal influence (McKay et al., 2022; Zhao et al., 2022), disruption of corneal extracellular matrix homeostasis or intracellular protein trafficking plausibly through miRNA due to rs2371597 polymorphism in *STON2* gene (Hosoda et al., 2020; Zhang et al., 2022) combined with age factor may help explain why the young males in our KC cohort are more susceptible to KC, highlighting the complex interplay of different biological and genetic factors may work together to affect the risk of KC.

Although *STON2* can be hypothesized to have an indirect role in KC development or progression through the speculated mechanism(s) discussed above, however, our study does not provide any functional or mechanistic evidence and future studies are needed to explore the consequences of the *STON2* rs2371597 polymorphism in KC pathogenesis. Besides, future research should also explore potential epistatic and gene-environment interactions to better understand how these polymorphisms influence KC risk in different settings.

The low allele frequency or absence of the rs11720822 [T] variant in *PDIA5* and the rs387907358 [T] and rs77542162 [G] variants in *WNT1* and *ABCA6*, respectively, among KC patients of Saudi origin suggests that these particular variants might not be significant risk factors for KC in Saudi individuals. Several factors might be attributed to the lack of significant associations for the polymorphisms rs11720822 in *PDIA5*, rs387907358 in *WNT1*, and rs77542162 in *ABCA6*. One key factor is population-specific genetic variability, where genetic variants can differ in frequency across populations due to historical and evolutionary influences. The results imply that these variants may be rare or absent in the Saudi population due to unique genetic drift or founder effects. As a result, the allelic frequencies and functional impacts of these polymorphisms might vary in the Saudi population compared to those observed in previous studies involving European or Asian cohorts (Ayub et al., 2014; Karolak et al., 2020b; Khawaja et al., 2019). This finding highlights the importance of considering population diversity when assessing genetic risk factors for diseases like KC.

The study has few limitations and the results require a cautious interpretation. The sample size examined in this study is relatively small, with even fewer numbers in subgroup analyses, particularly in the gender-stratified analysis. Nonetheless, our study exhibited power of 0.8 to detect significant associations between KC and rs2371597 *STON2* polymorphism for an OR of 2.0 and α (Type I error) of 0.05. Also, since ours is a tertiary care center there could be a referral or selection bias in the study and may not reflect the general Saudi population. Besides, the study lacks any mechanistic evidence to demonstrate the role of *STON2* polymorphism rs2371597 in KC. Therefore, further multi-center genetic research with larger and more diverse cohorts is essential to elucidate the genetic underpinnings of KC.

In conclusion, this study did not find strong associations between the investigated genetic polymorphisms and KC risk in the Saudi cohort. However, there is some evidence to suggest a gender-specific effect of rs2371597 polymorphism in the *STON2* towards increased risk of KC, particularly in men, indicating that genetic predispositions may interact with hormonal and/or environmental factors in this population. But, these findings are preliminary, and future research is needed to replicate these findings in large age- and sex-matched independent cohorts and diverse populations to elucidate the biological mechanisms through which this polymorphism might influence KC risk, particularly its gender-specific effects. Further investigation of the mechanisms underlying the complex interplay between age, genetics, and gender may help elucidate the overall disease risk profile. Finally, this study adds to the growing body of literature on the genetic basis of KC that may aid in developing targeted prevention and treatment strategies.

## Data Availability

The original contributions presented in the study are included in the article/supplementary material, further inquiries can be directed to the corresponding author.
